# Metabolic-epigenetic rewiring in rheumatoid arthritis: from pathogenic memory to precision restoration

**DOI:** 10.3389/fimmu.2026.1836618

**Published:** 2026-04-28

**Authors:** Shu Li, Lei Wan, Kun Wang, Xiaojun Zhang

**Affiliations:** 1 The First Affiliated Hospital of Anhui University of Chinese Medicine, Hefei, China; 2 Anhui University of Chinese Medicine, Hefei, China

**Keywords:** epigenetic reprogramming, immunometabolism, lactylation, rheumatoid arthritis, trained immunity

## Abstract

Rheumatoid arthritis (RA) is a systemic autoimmune disorder wherein sustained, drug-free remission remains an elusive clinical goal. Frequent disease flares upon treatment withdrawal indicate that conventional immunosuppression fails to eradicate a deeply ingrained “pathogenic memory.” In this Review, we provide a comprehensive framework illustrating how the hostile, nutrient-deprived synovial microenvironment acts as a metabolically restrictive microenvironment. Driven by “metabolic parasitism” and mitochondrial collapse, the massive accumulation of intermediate metabolites—most notably lactate, acetyl-CoA, and succinyl-CoA—transcends their traditional roles as bioenergetic waste to function as potent epigenetic regulators. We decode the emerging “PTM multiverse,” highlighting how aberrant lactylation, acetylation, and RNA modifications (ac4C) persistently rewire chromatin architecture and critical non-histone sensors (e.g., cGAS). Amplified by hyperactive acetyltransferases and the hypoxia-induced collapse of Sirtuin deacetylases, these modifications engrave resilient “epigenetic scars” that lock innate immune and stromal cells into highly destructive phenotypes via trained immunity. We further integrate this localized articular inflammation into a holistic meta-organ model, tracing disease origins to mucosal gene-environment interactions and detailing systemic regulation via the gut-microbiota-joint axis and chronobiological rhythms. Ultimately, we explore how deciphering these integrated networks translates into next-generation prognostic biomarkers (e.g., AMPAs and GlycA) and heralds a critical therapeutic paradigm shift—from transient immune blockade to precise metabolic-epigenetic restoration.

## 1 Introduction

Rheumatoid arthritis (RA) is a systemic, chronic autoimmune disorder characterized by progressive synovial hyperplasia, leukocyte infiltration, and irreversible osteocartilaginous destruction (1–3). Historically, the pathogenesis of RA has been defined by the breakdown of immune tolerance and the generation of autoantibodies, particularly anti-citrullinated protein antibodies (ACPAs) and a broader repertoire of anti-modified protein antibodies (AMPAs) (4–6). While the advent of biological and targeted synthetic disease-modifying antirheumatic drugs (DMARDs) has profoundly transformed clinical outcomes, a formidable therapeutic barrier persists. A substantial proportion of patients exhibit primary or secondary resistance, and sustained, drug-free remission remains an elusive goal (7, 8). The frequent recurrence of localized joint inflammation (flares) upon treatment withdrawal suggests that systemic immunosuppression fails to eradicate an underlying, deeply ingrained **“**pathogenic memory**”** residing within the joint microenvironment (9, 10).

To decode this pathogenic persistence, recent paradigm shifts have expanded the focus beyond conventional cytokine networks to the physicochemical realities of the inflamed joint. The rheumatoid synovium operates as a highly demanding ecological niche—a **“**bioreactor**”** characterized by severe hypoxia, aberrant angiogenesis, and profound nutrient deprivation (7, 11). Extensive multi-omics and functional analyses have revealed that infiltrating immune cells (such as CD4+ T cells and macrophages) and resident fibroblast-like synoviocytes (FLSs) are forced to undergo radical metabolic reprogramming to survive this hostile microenvironment (7, 12). This adaptation is hallmarked by impaired mitochondrial oxidative phosphorylation, reversed tricarboxylic acid (TCA) cycle dynamics, and a massive shift towards aerobic glycolysis (7, 13, 14). Consequently, this metabolic skewing leads to the relentless accumulation of intermediate metabolites, most notably lactate and acetyl-coenzyme A (acetyl-CoA), within the synovial fluid and intracellular compartments (11, 15).

Crucially, these accumulated metabolites are no longer viewed merely as bioenergetic byproducts or cellular waste. Instead, they act as potent signaling molecules that bridge cellular metabolism with chromatin architecture, orchestrating a profound metabolic-epigenetic rewiring (16, 17). Metabolites serve as direct substrates for a diverse array of protein post-translational modifications (PTMs)—a **“**PTM multiverse**”** that extends far beyond canonical citrullination to include acetylation, lactylation, succinylation, and malonylation (18, 19). Dysregulated acylations, orchestrated by imbalanced writers (e.g., histone acetyltransferases) and erasers (e.g., Sirtuins and histone deacetylases), physically alter histone tails and critical non-histone sensors, such as cyclic GMP-AMP synthase (cGAS) and ATP citrate lyase (20–23). For instance, the recently identified lactate-driven lactylation tightly locks macrophages and FLSs into aggressive, pro-inflammatory phenotypes, conferring apoptosis resistance and driving sustained tissue damage (24–26). It is this continuous loop of metabolic stress and epigenetic imprinting that inscribes persistent “epigenetic scars**”** on the innate immune and stromal compartments, fueling the chronicity of RA (9, 16).

Furthermore, this metabolic-epigenetic interplay is not confined to the local joint but is intricately connected to systemic homeostasis. Emerging evidence underscores the critical role of the gut-microbiota-joint axis, where microbiota-derived metabolites (such as short-chain fatty acids) and circadian dietary rhythms exert long-range epigenetic control over immune cell polarization (e.g., the Th17/Treg balance) (27–30). In this Review, we synthesize these latest breakthroughs to construct a comprehensive, multi-layered model of RA pathogenesis. By integrating conceptual visual models, we map the trajectory from environmental triggers and systemic metabolic shifts to the establishment of the synovial metabolic niche. We systematically decipher the roles of diverse PTMs—highlighting lactylation and Sirtuin-mediated deacetylation—in conferring pathogenic memory. Finally, we discuss how translating these immune-metabolic signatures into novel biomarkers (e.g., glycoprotein acetyls, GlycA) and targeted interventions (e.g., histone deacetylase [HDAC] and metabolic enzyme inhibitors) is paving the way for next-generation precision medicine (31–33), shifting the therapeutic horizon from broad immunosuppression to precise metabolic-epigenetic restoration (**Table 1**).

**Table 1:** The metabolic-epigenetic framework as an extension of the classical paradigm.

Core feature	Classical immunological paradigm	Emerging metabolic-epigenetic extension
Primary Disease Drivers	Aberrant cytokine networks and autoreactive lymphocyte clones.	Emphasizes the “PTM multiverse” driven by metabolic intermediates as critical amplifiers of these networks.
View of the Synovial Joint	Primary anatomical site of immune cell infiltration and structural damage.	An active, hostile “bioreactor” that metabolically forces infiltrating cells into altered epigenetic states.
Role of Intermediate Metabolites	Historically viewed as bioenergetic waste products (e.g., lactate causing local acidosis).	Recognized as potent signaling molecules and direct substrates for epigenetic reprogramming.
Nature of Autoantibodies	Established diagnostic markers and critical drivers of joint destruction (e.g., canonical ACPA).	Expanding to AMPAs acting as pathogenic bridges that directly alter osteoclast metabolism and complement pathways.
Cause of Disease Flare & Chronicity	Persistent residual immune cells and incomplete clearance of antigens.	Highlights resilient “epigenetic scars” and trained immunity locking cells into long-term pro-inflammatory memory.
Ultimate Therapeutic Goal	Continuous pharmacological immunosuppression to control systemic symptoms.	Augmenting immune blockade with precise Metabolic-Epigenetic Restoration (erasing pathogenic cellular memory).

## 2 The origins of RA: the triad of genetics, environment, and mucosal immunity

### 2.1 The pre-clinical window and the “Bermuda Triangle” model

The clinical manifestation of synovial inflammation represents merely the end-stage of a protracted pathophysiological process that often simmers for years, if not decades, prior to disease onset. The etiology of RA cannot be explained by simple Mendelian genetics; rather, it emerges from a highly orchestrated interplay conceptualized as a “Bermuda Triangle” of genetics, epigenetics, and autoimmunity (34). Within this framework, we emphasize environmental insults as major upstream triggers that initiate transient biochemical and metabolic disturbances, which are subsequently “fixed” into persistent autoimmune responses by genetic filters and epigenetic modifications. Understanding this pre-clinical window requires shifting our gaze from the articular cavity to the primary interfaces between the host and the external environment—the mucosal surfaces.

### 2.2 Mucosal surfaces as the primary sites for PTMs

**Figure 1:**
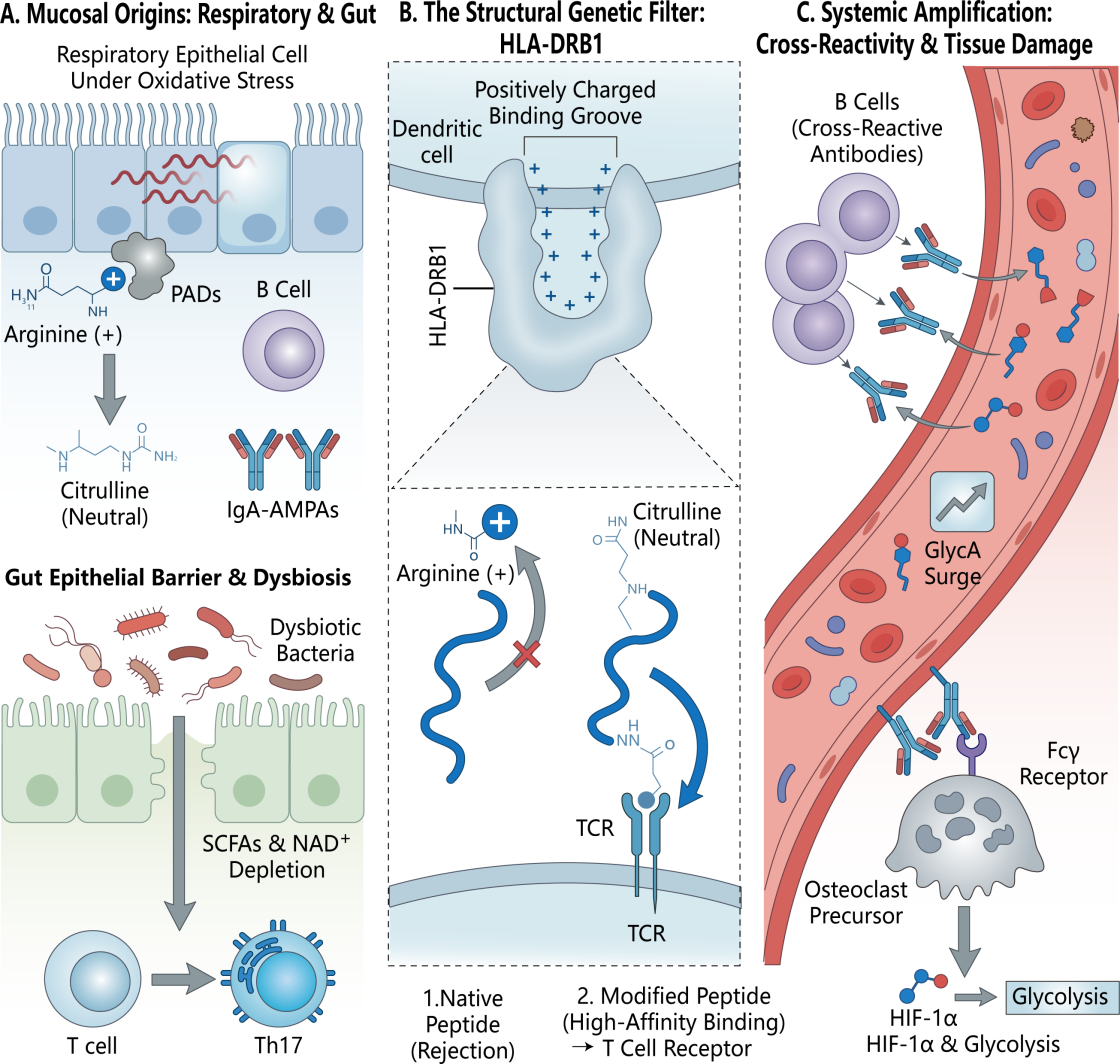
The multi-stage pathogenesis of rheumatoid arthritis: From mucosal triggers to systemic metabolic-epigenetic amplification. **(A)** Mucosal Origins: Environmental stressors (e.g., oxidative stress in the respiratory tract) activate peptidylarginine deiminases (PADs), driving the post-translational conversion of positively charged arginine to neutral citrulline. This local modification initiates the production of IgA-isotype anti-modified protein antibodies (AMPAs) by B cells. Concurrently, intestinal dysbiosis and barrier disruption lead to a depletion of essential metabolites (e.g., SCFAs and NAD^+^), tipping the local immune balance towards pathogenic Th17 cell activation. **(B)** The Genetic Filter (HLA-DRB1): The HLA-DRB1 shared epitope functions as a molecular funnel. Its positively charged peptide-binding groove electrostatically repels native, positively charged arginine peptides. Conversely, neutral citrullinated peptides fit precisely into this pocket, bypassing central tolerance and hyper-activating autoreactive T cells. **(C)** Systemic Amplification and Tissue Destruction: The breach of tolerance matures into a systemic storm characterized by highly cross-reactive autoantibodies and elevated circulating inflammatory metabolic biomarkers (e.g., GlycA). These autoantibodies directly engage Fcγ receptors on osteoclast precursors, triggering intracellular signaling cascades (including HIF-1α activation) that force a profound metabolic rewiring towards accelerated glycolysis, ultimately culminating in irreversible inflammatory bone resorption.

Mounting evidence designates mucosal tissues (e.g., lungs, periodontium, and gut) as the original primary sites where immune tolerance is breached (4, 34). Smoking, the most unequivocally established environmental risk factor for RA, exemplifies this mechanism. Chronic inhalation of cigarette smoke induces severe oxidative stress, hypoxia, and localized cellular apoptosis in the pulmonary mucosa (5, 35). This noxious microenvironment hyper-activates modifying enzymes (such as peptidylarginine deiminases, PADs) and disrupts local metabolite pools, triggering a massive surge in aberrant protein post-translational modifications (PTMs), including citrullination and acetylation (5, 34). Parallel to the lung, the periodontium and intestinal mucosa serve as critical sites for PTM generation and tolerance breach, as illustrated in **Figure 1**. Periodontal pathogens (e.g., Porphyromonas gingivalis) directly secrete PAD-like enzymes to generate citrullinated antigens, while intestinal dysbiosis compromises barrier integrity, causing a depletion of essential microbial metabolites (such as short-chain fatty acids, SCFAs) and NAD +. This mucosal disruption systematically tips the immune balance towards pathogenic Th17 cell activation. Consequently, these modified self-proteins act as “neo-antigens.” Recent comprehensive cohort analyses have revealed that smoking is predominantly associated with the generation of IgA-isotype anti-modified protein antibodies (AMPAs), specifically IgA-ACPAs and IgA-anti-acetylated protein antibodies (AAPA) (35). The prominent IgA signature strongly corroborates the hypothesis that the initial autoantibody repertoire matures within the mucosal-associated lymphoid tissues before disseminating systemically (4, 35).

### 2.3 The HLA-shared epitope: a molecular funnel for PTM peptides

The generation of mucosal neo-antigens alone is insufficient to trigger systemic autoimmunity without a permissive genetic background. The most formidable genetic risk factors for RA are the HLA-DRB1 alleles containing the “Shared Epitope” (SE). The structural biology of the HLA-SE provides a mechanistic explanation for its synergistic interaction with environmental PTMs. The antigen-binding groove of the SE is characterized by a positive charge, which exhibits poor affinity for native, positively charged arginine residues. However, when environmental stress drives the conversion of arginine to neutral citrulline, or lysine to acetyl-lysine, these PTM-altered peptides fit perfectly into the HLA-SE pocket (4, 34). This high-affinity presentation effectively bypasses central tolerance, activating autoreactive CD4+ T cells. Strikingly, recent meta-analyses demonstrate that the gene-environment (G×E) interaction between smoking and HLA-SE alleles is exclusively significant in patients harboring both IgG and IgA isotypes of ACPAs, underscoring that the fatal convergence of genetic predisposition and environmental PTMs occurs primarily at mucosal sites (35). Furthermore, B cells recognizing these modified antigens exhibit extreme cross-reactivity towards various PTMs (citrullination, carbamylation, acetylation) due to the recognition of “shared motifs,” facilitating a dynamic and highly diversified autoantibody repertoire (5, 36) (**Figure 1**).

### 2.4 Systemic metabolomic buffering: the role of dietary environment

Beyond localized mucosal insults, macro-environmental factors such as dietary patterns exert a profound systemic influence by modulating the body**’**s baseline metabolomic and epigenetic tone. Large-scale prospective studies, such as those utilizing the UK Biobank, have illuminated that adherence to specific dietary regimens (e.g., the EAT-Lancet diet) significantly mitigates the risk of incident RA (37). Remarkably, over one-third of this protective effect is mediated through the remodeling of the host’s systemic metabolomic signature (37). Health-promoting diets actively lower circulating inflammatory metabolic markers, such as glycoprotein acetyls (GlycA), while simultaneously enriching protective lipid species like omega-3 polyunsaturated fatty acids (e.g., DHA). This systemic metabolic shift serves as an epigenetic “buffer,” capable of counteracting the inflammatory risks conferred by specific genetic variants—such as Beta-2 Microglobulin (B2M) and Solute Carrier Family 30 Member 4 (SLC30A4). These specific loci, implicated in systemic immune regulation and zinc transport respectively, were identified in large-scale cohorts as being significantly modulated by diet-derived metabolomic signatures, demonstrating how macro-environmental nutrition can phenotypically silence innate genetic susceptibility through potent gene-diet interactions (37). Thus, while localized mucosal stress initiates the autoimmune process, the systemic metabolomic landscape dictates whether this early disruption resolves or escalates into clinical rheumatoid disease (**Table 2**).

**Table 2:** The “Bermuda Triangle” of RA pre-clinical origins.

Trigger/factor	Category	Primary anatomical site	Pathological & metabolic consequence in pre-clinical RA	Ref.
Smoking	Environmental Insult	Pulmonary Mucosa	Induces oxidative stress/hypoxia; hyper-activates PADs; triggers the generation of IgA-isotype AMPAs (e.g., IgA-ACPA).	(5, 35)
HLA-DRB1 “Shared Epitope”	Genetic Susceptibility	Systemic (Lymphatic)	Provides a positively charged binding groove that preferentially binds neutral, PTM-altered peptides, bypassing central tolerance.	(4, 34)
EAT-Lancet Diet	Systemic Buffer (Nutrition)	Systemic Circulation	Remodels the host’s metabolomic signature; lowers inflammatory GlycA and enriches protective polyunsaturated fatty acids.	(37)
Eggerthella lenta	Gut Pathobiont (Dysbiosis)	Intestinal Microbiome	Depletes systemic amino acids and NAD+ levels; drives a senescent-like inflammatory state that augments autoantibody production.	(38)
Low-Protein Diet/Botanical Regimens	Systemic Buffer (Nutrition/Microbiome)	Systemic Circulation & Gut	Activates the NRF2/SIRT3 antioxidant axis to mitigate M1 polarization; enriches SCFA-producing probiotics to fortify mucosal barriers.	(39, 40)

## 3 The synovial niche: metabolic parasitism and resource competition

### 3.1 The synovial “bioreactor” and metabolic monopolization

Upon breaching systemic tolerance and migrating into the joint, autoreactive immune clones encounter a remarkably hostile physicochemical microenvironment. The hyperplastic rheumatoid synovium functions as an aberrant “bioreactor” characterized by profound hypoxia, high mechanical stress, and severe nutrient deprivation (7). Within this crowded ecological niche, cellular survival dictates a radical shift in bioenergetics. Activated resident fibroblast-like synoviocytes (FLSs) and pro-inflammatory macrophages undergo a “Warburg-like” metabolic reprogramming, heavily skewing their metabolism toward aerobic glycolysis. This metabolic monopolization allows these dominant cell types to consume local glucose at extraordinarily high rates, effectively depleting the synovial fluid of its primary carbon source (7). Drawing a conceptual analogy from cancer immunology—where tumor cells metabolically starve infiltrating lymphocytes (41)—this asymmetric nutrient competition in the RA synovium functions as a form of “metabolic parasitism”. Consequently, this relentless resource monopolization forces infiltrating adaptive immune cells to adopt alternative, often suboptimal, metabolic survival strategies that inadvertently lock them into pathogenic phenotypes.

### 3.2 T cell starvation, mitochondrial collapse, and TNF super-production

Deprived of glucose, synovial CD4+ T cells are forced to rely on amino acids—primarily glutamine and glutamate—as their predominant fuel sources (7). This forced metabolic adaptation precipitates a severe decline in mitochondrial performance. RA T cells exhibit fundamentally defective mitochondrial DNA repair and diminished ATP production, accompanied by an anomalous reversal of the tricarboxylic acid (TCA) cycle (7, 13). A critical consequence of this mitochondrial failure is the profound shortage of mitochondrial aspartate (13). This specific amino acid deficiency disrupts the malate-aspartate shuttle and cytosolic NAD+ regeneration, directly causing ADP-deribosylation of the endoplasmic reticulum (ER) stress sensor glucose-regulated protein 78/binding immunoglobulin protein (GRP78/BiP) (13). Relieved of its regulatory constraints, the ribosome-rich ER membrane undergoes massive expansion, hyper-accelerating the co-translational translocation and biogenesis of transmembrane tumor necrosis factor (TNF). Through this mitochondria-to-ER retrograde signaling axis, bioenergetically starved T cells are transformed into highly potent “TNF super-producers.” Importantly, this metabolic competition establishes a mutually reinforcing vicious cycle: the massive release of TNF-α by these nutrient-deprived T cells acts as a potent paracrine signal that further hyper-activates the glycolytic machinery and HIF-1α signaling in adjacent FLSs and macrophages. This continuous cross-talk perpetually deepens the localized nutrient deficit, ensuring neither cell population escapes the pathogenic state (7, 13). Furthermore, dysregulated kinases such as mammalian STE20-like kinase 1 (MST1) further exacerbate this organelle crisis in synoviocytes by inhibiting the SIRT3/mTOR pathway, impairing mitophagy and promoting pathological mitochondrial fission (14).

### 3.3 The intercellular shuttling of metabolites and NET-driven lipogenesis

The consequences of this dysregulation extend far beyond single-cell dysfunction. The hyper-glycolytic rate of FLSs and macrophages generates excessive quantities of extracellular lactate, drastically acidifying the synovial fluid. Rather than merely acting as an inert byproduct, lactate operates as a potent paracrine signaling molecule (lactate shuttling). Uptake of exogenous lactate by adjacent endothelial cells suppresses SIRT1 activity, unleashing the acetylation of p53 and p65 to drive pathological angiogenesis (pannus formation) (15, 42). Simultaneously, high lactate and extracellular acidification trigger acid-sensing ion channels (e.g., ASIC1a) on chondrocytes, precipitating excessive mitophagy and PANoptosis, thereby accelerating irreversible cartilage degradation (15, 43). Furthermore, the localized inflammatory milieu is heavily infiltrated by neutrophils that undergo NETosis. Neutrophil extracellular traps (NETs) directly stimulate ATP citrate lyase (ACLY) in FLSs, forcibly redirecting glucose/lipid metabolism toward *de novo* lipogenesis (DNL) and markedly expanding the intracellular acetyl-CoA pool (44).

**Figure 2:**
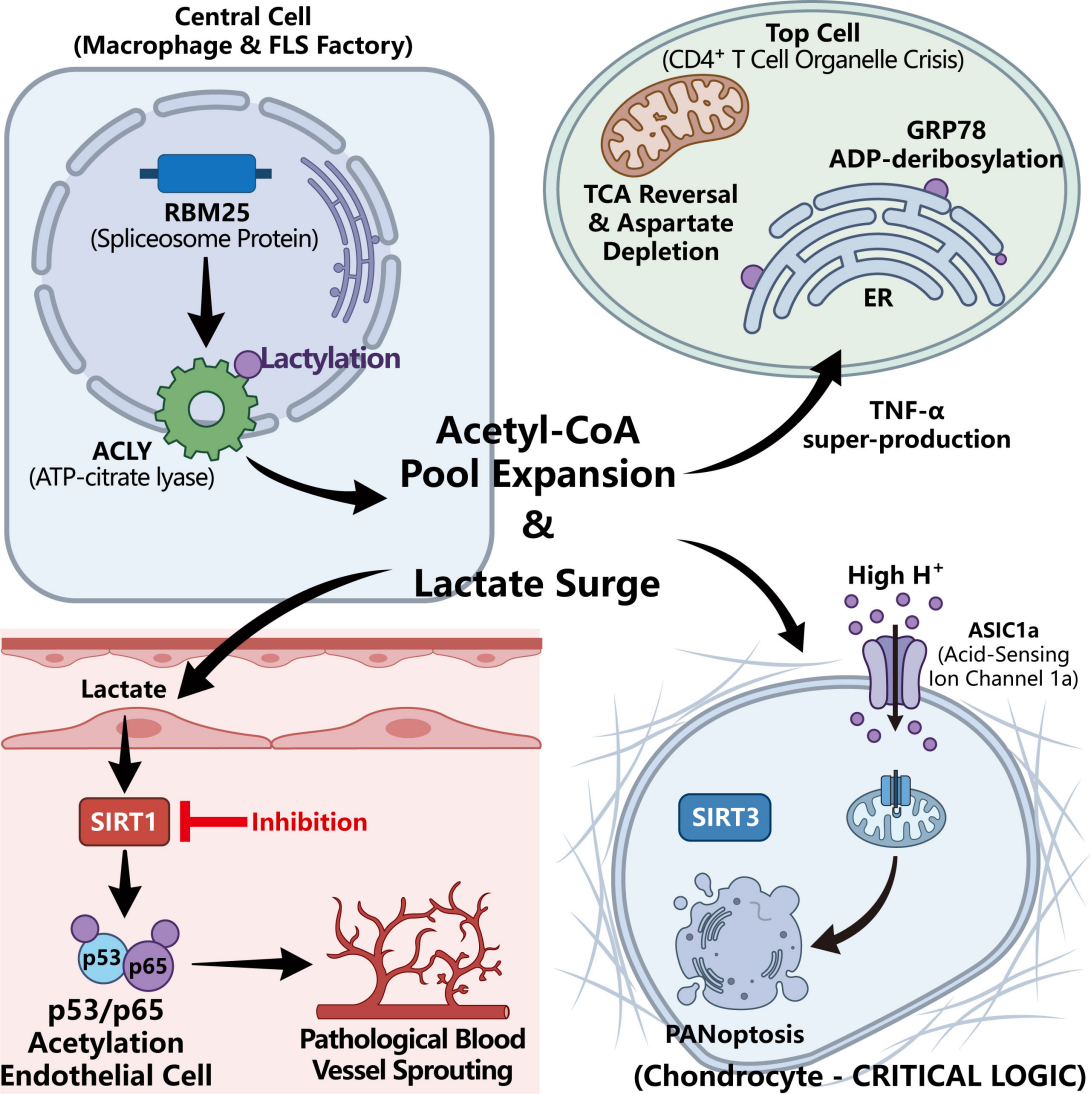
Metabolic parasitism and intercellular signaling networks within the rheumatoid synovial bioreactor. The hypoxic and nutrient-deprived synovial microenvironment forces diverse resident and infiltrating cells into a highly intertwined metabolic-epigenetic web. (Central) Macrophage & FLS Factory: Driven by severe inflammation, the downregulation of the spliceosome protein RBM25 leads to aberrant ACLY splicing and robust ACLY lactylation. This heavily rewires metabolism, triggering a massive expansion of the intracellular acetyl-CoA pool and a massive lactate surge into the extracellular space. (Top) CD4+ T Cell Organelle Crisis: Deprived of local glucose, starved T cells undergo tricarboxylic acid (TCA) cycle reversal, leading to a profound depletion of mitochondrial aspartate. This specific deficit triggers endoplasmic reticulum (ER) stress via GRP78 deribosylation, ultimately transforming T cells into highly potent TNF-α super-producers. (Bottom Left) Endothelial Cell Reprogramming: Through paracrine lactate shuttling, exogenous lactate enters adjacent endothelial cells and decisively suppresses the epigenetic eraser SIRT1. The resulting hyper-acetylation of p53 and p65 unleashes aggressive, pathological blood vessel sprouting (pannus formation). (Bottom Right) Chondrocyte Destruction: The extracellular acidification (H^+^ surge) driven by massive lactate efflux persistently activates acid-sensing ASIC1a channels on chondrocytes. This ionic stress impairs the SIRT3 protective pathway, precipitating irreversible joint degradation via PANoptosis.

Beyond soluble metabolites, intercellular communication within the synovial bioreactor is profoundly mediated by extracellular vesicles. Fibroblast-like synoviocytes (FLSs) release inflammatory exosomes that directly penetrate adjacent macrophages, forcibly shifting their metabolic profile toward hyper-glycolysis and M1 polarization. This exosome-driven metabolic rewiring subsequently amplifies pathological histone lactylation (e.g., H3K56la), creating a self-sustaining loop of synovial inflammation that can be effectively intercepted by targeted natural compounds such as geniposidic acid (45). Concurrently, the hypoxic and metabolically deprived environment upregulates cytoskeleton-associated proteins, such as Vav3, which orchestrates aggressive cellular invasion and supports pathological neoangiogenesis (pannus formation), adding a critical spatial dimension to the tumor-like expansion of the synovium (46). Furthermore, this localized metabolic stress is intimately linked to systemic lipid metabolism. Inflammatory stimuli compel adipocytes to synthesize aberrant lipid mediators, establishing an “adipocyte-macrophage” metabolic-immune feedback loop. Suppressing this feedback through metabolic sensors like PPAR-γ has emerged as a crucial strategy to prevent systemic metabolic exhaustion from continuously fueling joint inflammation (47). Crucially, as detailed in **Figure 2**, this intense intercellular stress is internally relayed within macrophages via the downregulation of the spliceosome protein RBM25, a key upstream modulator that redirects ACLY splicing and robustly expands the intracellular acetyl-CoA pool (20) (**Figure 2**).

### 3.4 Setting the stage: metabolites as epigenetic substrates

In summary, the rheumatoid synovial niche is not merely a passive anatomical site of inflammation, but a highly active metabolic battleground. The relentless resource competition, mitochondrial dysfunction, and pervasive intercellular metabolite shuttling ultimately result in the massive intracellular accumulation of specific intermediates—most notably lactate, acetyl-CoA, and citrate (7, 15, 44). Escaping the confines of the mitochondria and cytoplasm, these overflowing high-energy metabolites permeate the nucleus, serving as the direct biochemical substrates for modifying chromatin. This precise intersection of metabolic overflow and nuclear accessibility may create a permissive context for the extensive epigenetic remodeling that contributes to the chronicity of RA (**Table 3**).

**Table 3:** Metabolic parasitism and organelle dysfunction in the synovial niche.

Target cell type	Microenvironmental stressor	Altered metabolic/subcellular pathway	Downstream molecular & pathological consequence	Ref.
CD4+ T Cells	Glucose deprivation	Mitochondrial Collapse: Defective mtDNA repair, TCA cycle reversal, and aspartate shortage.	Triggers ER stress (GRP78/BiP deribosylation) and massive expansion of ER membranes, creating TNF super-producers.	(7, 13)
Fibroblast-like Synoviocytes (FLSs)	Hypoxia & NETosis	Glycolytic Monopolization & DNL: Upregulation of aerobic glycolysis and ACLY-mediated lipogenesis.	Generates excessive amounts of lactate and acetyl-CoA, fueling massive downstream epigenetic modifications.	(7, 25, 44)
Pro-inflammatory Macrophages	Lactic acidosis & Hypoxia	Spliceosome Dysfunction: RBM25 downregulation causes aberrant Acly splicing (Long isoform).	Intensive lactylation of ACLY L alters its substrate affinity, fueling a positive feedback loop of glycolysis and M1 polarization.	(20)
Chondrocytes	Synovial Extracellular Acidification	Acid-sensing Activation: Activation of ASIC1a channels.	Disrupts SIRT3 mitochondrial translocation, inducing excessive mitophagy and irreversible PANoptosis of cartilage.	(15, 43)
Endothelial Cells	Paracrine Lactate Shuttling	Deacetylase Suppression: Inhibition of SIRT1 activity by exogenous lactate uptake.	Unleashes the acetylation of p53 and NF-κB p65, aggressively promoting pathological angiogenesis (pannus formation).	(15, 42)
Macrophages	FLS-derived Inflammatory Exosomes	Exosome-driven Glycolytic Rewiring: Forced shift toward hyper-glycolysis.	Amplifies pathological histone lactylation (H3K56la), creating a self-sustaining loop of M1 polarization and synovial inflammation.	(45)
Adipocytes & Macrophages	Localized Metabolic Stress	Aberrant Lipid Mediator Synthesis: Disrupted fat anabolism and altered PPAR-γ signaling.	Establishes an “adipocyte-macrophage” metabolic-immune feedback loop that continuously fuels joint inflammation.	(47)

## 4 Lactylation: rewiring the epigenetic landscape

### 4.1 The conceptual leap: from metabolic waste to epigenetic regulator

For decades, the profound lactic acidosis characteristic of the rheumatoid joint was considered merely a toxic byproduct of the hyper-glycolytic “Warburg” effect, contributing passively to tissue damage and pain. However, the groundbreaking discovery of protein lactylation—a novel post-translational modification wherein lactate is covalently attached to lysine residues (Kla)—has fundamentally redefined the pathogenic hierarchy of RA (17, 24). Lactate is no longer viewed as terminal metabolic waste; rather, it functions as a potent, master epigenetic regulator. By serving as a direct substrate for chromatin remodeling, the excessive pool of intracellular lactate physically bridges the hyperactive glycolytic flux with the transcriptional machinery, translating the metabolic distress of the synovial niche into durable, pro-inflammatory “epigenetic scars” (48).

### 4.2 Histone lactylation and the apoptosis resistance of synovial fibroblasts

In resident fibroblast-like synoviocytes (FLSs), elevated lactate actively drives robust histone lactylation, endowing these cells with a highly aggressive, tumor-like phenotype. Specifically, lactate stimulation dramatically upregulates histone H3 lysine 18 lactylation (H3K18la) orchestrated by the p300 acetyltransferase. This specific epigenetic mark recruits and activates methyltransferase 1 (METTL1), which in turn mediates the m7G modification of NeuroD1 mRNA, significantly enhancing its stability. The accumulated NeuroD1 ultimately boosts glutathione peroxidase 4 (GPX4) transcription, conferring profound ferroptosis resistance upon FLSs and enabling their unchecked hyperplasia within the destructive pannus (25). Concurrently, lysine acetyltransferase 8 (KAT8) acts as a “writer” to deposit H3K9 lactylation (H3K9la) on the promoters of pro-inflammatory cytokines and matrix metalloproteinases (MMPs), locking FLSs in a catabolic state—a pathological sequence that can be therapeutically intercepted by targeted KAT8 inhibitors like gastrodin (49). Additionally, modulation of H3K18la and H3K27la has been shown to be critical in dictating FLS and macrophage behavior, further highlighting the ubiquitous nature of these marks in synovial pathology (26).

The epigenetic impact of the lactate storm extends far beyond the synovial stroma and innate immune cells; it decisively disrupts bone homeostasis. The accumulation of lactate serves as a powerful microenvironmental cue that dynamically regulates the epigenetic landscape of bone-resorbing cells. Aberrant lactylation levels alter the transcriptional equilibrium between osteoblasts and osteoclasts, severely accelerating osteoclast differentiation and driving the irreversible osteocartilaginous destruction characteristic of late-stage RA (50).

### 4.3 RNA splicing, ACLY lactylation, and macrophage polarization

The lactylation network extends beyond the stroma to dictate the fate of innate immune cells through intricate positive feedback loops. In RA macrophages, chronic inflammation triggers the downregulation of the spliceosome component RBM25. This deficiency forces the aberrant alternative splicing of ATP citrate lyase (Acly) pre-mRNA, overwhelmingly increasing the expression of its short isoform while subjecting the long isoform (Acly L) to intense lactylation at specific lysine residues (20). This targeted lactylation drastically alters ACLY’s affinity for metabolic substrates, supercharging acetyl-CoA production and reinforcing glycolytic reprogramming. Consequently, this continuous autocrine lactate supply sustains heavy epigenetic remodeling, persistently trapping macrophages in a hyper-activated, tissue-destructive M1-like polarization state. Furthermore, high-resolution single-cell RNA sequencing reveals that this profound lactylation signature is also strongly enriched in specific autoantibody-producing plasma cell subsets, correlating tightly with immune checkpoint expression and disease severity (51).

### 4.4 Non-histone lactylation: the cGAS-STING amplifier

Beyond histones and metabolic enzymes, the “PTM multiverse” in RA encompasses the lactylation of critical non-histone signaling sensors, most notably the cytosolic DNA sensor cyclic GMP-AMP synthase (cGAS). In the hypoxic, DNA-rich synovial microenvironment, cell death releases abundant mitochondrial DNA (mtDNA), which typically activates the cGAS-STING pathway to induce type I interferons. Under physiological conditions, cGAS is strictly regulated by MARCHF5-mediated ubiquitination and subsequent degradation to prevent runaway inflammation. However, the RA lactate storm competitively drives the lactylation of cGAS at essential lysine residues. This bulky lactyl group creates severe steric hindrance, physically shielding cGAS from E3 ubiquitin ligase recognition (22, 52). By effectively disabling its degradation switch, lactylation persistently sustains cGAS signaling, amplifying a continuous, sustained inflammatory and interferon cascade. Thus, targeting the lactylation of non-histone sensors presents a highly specific vulnerability to attenuate the core autoimmune response.

## 5 The acetylation storm, sirtuin collapse, and the PTM multiverse

### 5.1 The acetylation storm and hyperactive “writers”

Parallel to the lactate-driven epigenetic rewiring, the metabolic bottleneck in the rheumatoid joint provokes a massive accumulation of intracellular acetyl-CoA. As the obligate acyl donor for lysine acetyltransferases (KATs), this overflowing acetyl-CoA reservoir fuels a pervasive “acetylation storm” across the chromatin landscape. In pro-inflammatory macrophages, the hyperactive “writer” KAT2A explicitly utilizes this metabolic surplus to deposit activating histone H3 lysine 9 acetylation (H3K9ac) marks on the promoters of Il1b and Nlrp3. This modification forcefully drives NLRP3 inflammasome assembly while simultaneously repressing NRF2-mediated antioxidant defenses (53). Similarly, within the hostile synovial stroma, the p300/CBP acetyltransferase complex comprehensively rewires the transcriptional network of FLSs. By hyper-acetylating specific stress-response gene loci, p300 immobilizes FLSs in a state of heightened resistance to hypoxia and oxidative stress, thereby sustaining their destructive, invasive properties (54).

### 5.2 The collapse of epigenetic guardians: the sirtuin family

Concomitant with the hyperactivity of epigenetic writers, the rheumatoid niche witnesses a profound impairment of the epigenetic “erasers”—specifically, the NAD+-dependent class III histone deacetylases known as Sirtuins (21, 55). The mitochondrial collapse and severe NAD+ depletion within the hypoxic joint directly dismantle Sirtuin enzymatic activity (13). The downregulation of SIRT1 in endothelial cells unleashes the acetylation of p53 and NF-κB p65, aggressively promoting pathological angiogenesis and pannus expansion (42). In CD4+ T cells, SIRT3 deficiency critically impairs PFKFB3-driven glycolysis, inducing severe metabolic inflexibility and rendering them highly arthritogenic (56). Furthermore, the dysregulation of SIRT4 in the autoimmune setting fails to deacetylate the DNA sensor cGAS, abnormally enhancing its affinity for double-stranded DNA and further fueling the relentless type I interferon signaling cascade (57). This systemic Sirtuin collapse definitively tips the epigenetic scale toward sustained inflammation.

The collapse of the Sirtuin epigenetic guardians in the rheumatoid niche is not solely a consequence of NAD+ depletion but is tightly orchestrated at the post-transcriptional level by complex non-coding RNA (ncRNA) networks. In RA FLSs, the pathological downregulation of protective circular RNAs (e.g., hsa_circ_0044235 and circ-Sirt1) and long non-coding RNAs (like GAS5), coupled with the overexpression of specific microRNAs (such as miR-222-3p and miR-135b-5p), creates a “sponge effect” that robustly blocks SIRT1 translation (58–60). This precise ncRNA-mediated blockade unleashes the hyper-acetylation of NF-κB, vigorously driving cell pyroptosis and massive cytokine release. Beyond SIRT1, other Sirtuin family members constitute essential, yet vulnerable, metabolic defense lines. SIRT3 is critical for mitigating mitochondrial oxidative stress via the SOD2/ROS pathway to prevent pro-inflammatory macrophage polarization (40), while SIRT6 synergizes with AMPK signaling to suppress lipid-induced endoplasmic reticulum stress (61). Ultimately, the ubiquitous disruption of the HAT/HDAC equilibrium transcends local articular boundaries, serving as a core epigenetic driver for systemic cartilage degradation and aberrant cell survival across various rheumatic diseases (62).

### 5.3 Beyond chromatin: the RNA acetylation frontier

Crucially, the metabolic-epigenetic axis tightly controls the inflammatory rheostat not only at the transcriptional level via chromatin remodeling, but also at the post-transcriptional level through epitranscriptomics. Extending the epigenetic frontier beyond traditional DNA-associated histones, recent breakthroughs have unveiled that the acetyl-CoA surge also serves as the direct substrate for RNA N4-acetylcytidine (ac4C) modifications (63). In rheumatoid FLSs, the RNA acetyltransferase NAT10 is significantly upregulated, orchestrating a profound ac4C enrichment on specific pathogenic transcripts. Functioning as a crucial post-transcriptional bridge between acetyl-CoA abundance and phenotypic outcomes, the metabolic surge directly fuels NAT10 activity. Current evidence in RA suggests that, rather than relying strictly on canonical chromatin “reader” proteins, NAT10-mediated ac4C modification acts primarily by hyper-stabilizing target transcripts, such as PTX3 mRNA, and drastically enhancing their translational efficiency. This RNA-level epigenetic fortification ensures continuous pathogenic protein production and directly promotes synovial aggression, orchestrating a dense infiltration of immune cells into the joint (64). Thus, the metabolic-epigenetic axis tightly controls the inflammatory rheostat at both the transcriptional (DNA) and post-transcriptional (RNA) levels.

### 5.4 The PTM multiverse and the imprinting of immune memory

The metabolic dysregulation in RA generates a diverse array of short-chain acyl-CoAs, expanding the epigenetic landscape into a sprawling “PTM multiverse.” Scientifically, we define this “multiverse” as the dynamic coexistence, competition, and functional crosstalk among diverse metabolite-driven PTMs. On chromatin, this crosstalk manifests prominently between lactylation and acetylation; because both modifications often target identical lysine residues on histone tails and partially share writer enzymes (e.g., the p300 acetyltransferase), the relative availability of lactate versus acetyl-CoA tightly dictates the specific epigenetic output. Beyond these primary marks, the multiverse encompasses other critical acylations. For instance, mitochondrial TCA cycle defects in RA T cells lead to the toxic accumulation of succinyl-CoA, which acts as a direct substrate for the massive succinylation of the transcription factor BRD2. By introducing a bulky negatively charged group that alters protein conformation, this modification represses proteasomal degradation pathways (Psmb5), allowing the accumulation of the Hobit transcription factor and biasing these cells toward a highly pathogenic, tissue-resident memory T cell (Trm) phenotype (65). Concurrently, aberrant lipid metabolism yields excess malonyl-CoA. This intermediate acts as an independent epigenetic substrate, installing a unique malonylation signature on cytoskeletal and functional neutrophil proteins that hyper-activates destructive NETosis (66).

**Figure 3:**
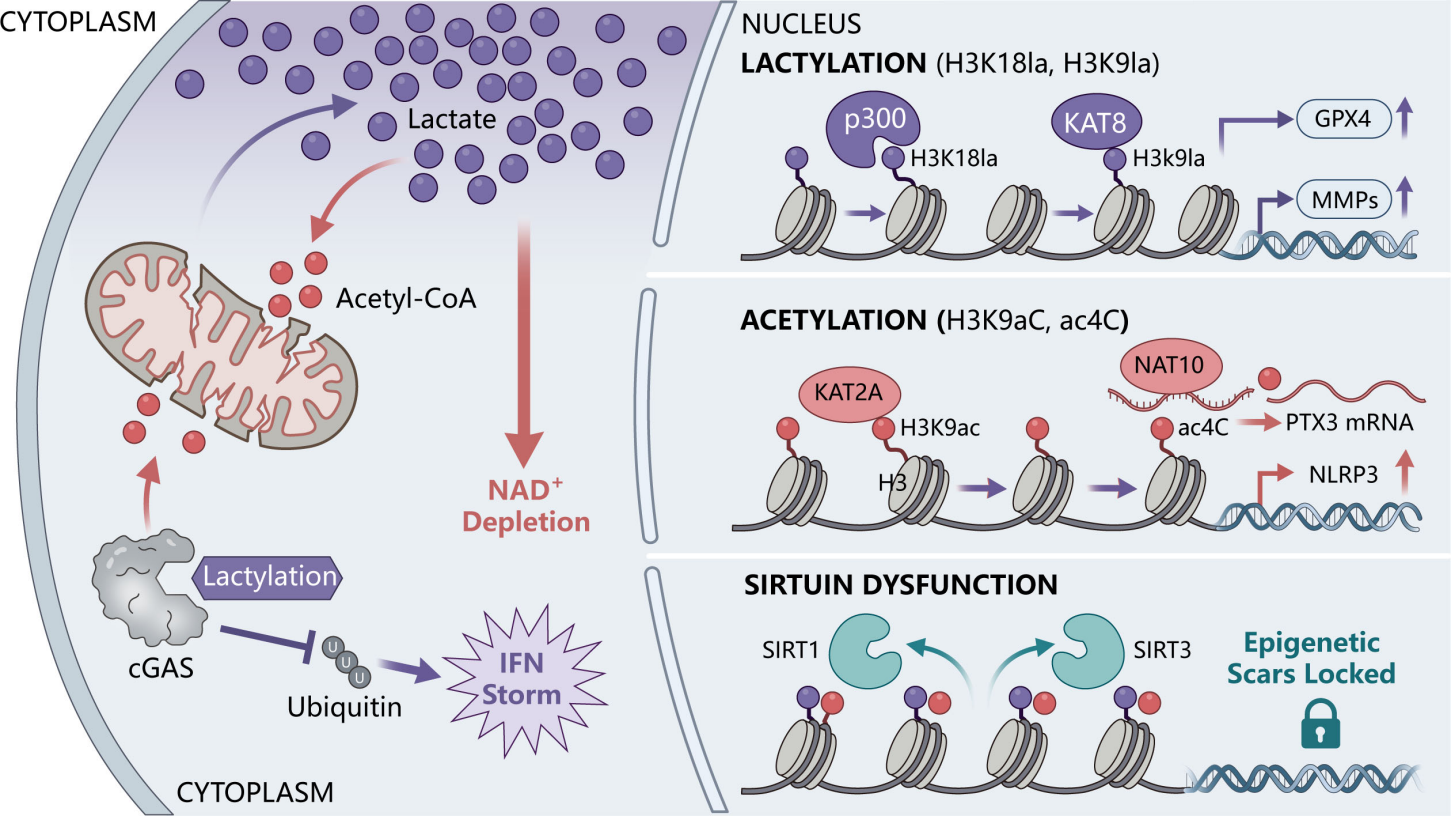
The “PTM Multiverse” orchestrates pathogenic memory and epigenetic locking in the rheumatoid synovium. The hostile synovial microenvironment functions as a metabolic crucible, driving profound intracellular accumulation of lactate and acetyl-CoA alongside severe NAD^+^ depletion. (Left) In the cytoplasm, the lactate surge directly lactylates the DNA sensor cGAS, causing steric hindrance that blocks MARCHF5-mediated ubiquitination, thereby fueling an sustained Type I interferon storm. (Right) Within the nucleus, these overflowing metabolites serve as substrates for a dysregulated “PTM multiverse.” Driven by overactive “writers,” the lactylation pathway (via p300 and KAT8) deposits H3K18la and H3K9la marks to upregulate GPX4 and MMPs, conferring ferroptosis resistance and driving tissue destruction. Concurrently, the acetylation pathway (via KAT2A and NAT10) utilizes acetyl-CoA to install H3K9ac and RNA ac4C modifications, hyperactivating the NLRP3 inflammasome and stabilizing PTX3 mRNA. Crucially, the severe NAD^+^ depletion induces the functional collapse of epigenetic “erasers” (SIRT1/SIRT3). The failure of deacetylation mechanisms leaves these aberrant modifications unopposed, persistently locking resident stromal and immune cells into highly destructive phenotypes via trained immunity (epigenetic scars).

Ultimately, this fierce competition among lactylation, acetylation, succinylation, and malonylation for specific lysine residues weaves a persistent “epigenetic scar” on the genome. This phenomenon, termed trained immunity (or innate immune memory), physically hardwires macrophages and FLSs into a poised, hyper-reactive state (9, 67). It explains the fundamental clinical conundrum of RA: even when transient inflammatory triggers are cleared or traditional immunosuppressants are applied, the entrenched epigenetic architecture ensures that the pathogenic memory remains dormant yet highly stable, primed to facilitate ignite recurrent disease flares (**Figure 3**) (**Table 4**).

**Table 4:** The “PTM multiverse” and epigenetic dysregulation in RA.

Upstream metabolite/status	Epigenetic mechanism (PTM)	Modulating enzyme	Target substrate	Pathological consequence in RA synovium	Ref.
Lactate (Accumulation)	Histone Lactylation	p300/KAT8	H3K18la/H3K9la	Activates GPX4/MMPs, conferring ferroptosis resistance and driving pro-inflammatory transcription in FLSs.	(25, 49)
Lactate (Accumulation)	Non-histone Lactylation	Unknown	cGAS (DNA sensor)	Causes steric hindrance, shielding cGAS from ubiquitination and driving sustained Type I IFN storms.	(22, 52)
Acetyl-CoA (Accumulation)	Histone Acetylation	KAT2A/p300	H3K9ac/Promoters	Hyper-activates the Nlrp3 inflammasome; immobilizes FLSs in a state of heightened resistance to hypoxia.	(53, 54)
Acetyl-CoA (Accumulation)	RNA Acetylation (ac4C)	NAT10	PTX3 mRNA	Enhances mRNA stability and translational efficiency, expanding the frontier to epitranscriptomics.	(64)
Succinyl-CoA (Accumulation)	Protein Succinylation	Unknown	BRD2	Represses proteasomal degradation (Psmb5), locking T cells into a pathogenic tissue-resident memory (Trm) state.	(65)
NAD+ (Severe Depletion)	Sirtuin Collapse (Failure of Deacetylation)	SIRT1/SIRT3/SIRT4	p65, cGAS, PFKFB3	Eraser failure tips the epigenetic scale, causing metabolic inflexibility, angiogenesis, and sustained innate immune activation.	(42, 56, 57)
ncRNA Dysregulation (e.g., GAS5↓, miR-222-3p↑)	Sirtuin 1 Translational Blockade	SIRT1 (Eraser Collapse)	NF-κB	Releases NF-κB hyper-acetylation, vigorously driving cell pyroptosis and massive pro-inflammatory cytokine release.	(58–60)
Epigenetic Recognition (Acetyl-marks)	Reading of Acetylated Histones	BET proteins (Readers)	Chromatin/Promoters	Recognizes acetyl-marks to promote the transcription of matrix-degrading enzymes and accelerate osteoclastogenesis.	(68)

## 6 Systemic control: the gut-joint axis and chronobiological metabolic rhythms

### 6.1 Dysbiosis and the depletion of systemic epigenetic guardians

While the synovial microenvironment generates localized pathological metabolites (e.g., lactate) that drive hyper-acetylation and lactylation, the systemic epigenetic tone is heavily governed by a distant metabolic factory: the gut microbiota. In RA, the intestinal microbiome undergoes a characteristic dysbiotic shift that deprives the host of vital epigenetic guardians. Shotgun metagenomic sequencing has revealed a profound deficiency in butyrate-producing species alongside an overwhelming expansion of butyrate-consuming bacteria in patients with new-onset RA (28). Concurrently, in preclinical animal models, the expansion of pathogenic pathobionts, such as Eggerthella lenta, has been shown to promote a systemic decline in amino acids and the critical metabolic cofactor NAD+, culminating in a “senescent-like” systemic inflammatory state that exacerbates preclinical autoantibody production and joint damage (38). This microbial metabolic collapse significantly reduces the systemic availability of key short-chain fatty acids (SCFAs), particularly butyrate, directly leaving the synovial epigenetic landscape vulnerable to pro-inflammatory remodeling.

### 6.2 SCFAs as long-range epigenetic modulators of T cell fate

Microbiota-derived SCFAs—predominantly butyrate and propionate—serve as critical inter-organ messengers that remote-control the Th17/Treg equilibrium within the joint (69). To exert these systemic effects, SCFAs must first cross the intestinal epithelial barrier. This transcellular transport is actively mediated by specific solute carriers, notably monocarboxylate transporter 1 (MCT1/SLC16A1) and sodium-coupled monocarboxylate transporter 1 (SMCT1/SLC5A8). Upon uptake into the portal and systemic circulation, these metabolites disseminate to remote tissues and penetrate immune cellsto orchestrate T cell fate through a dual-pronged molecular mechanism. Epigenetically, butyrate functions as a potent, naturally occurring histone deacetylase (HDAC) inhibitor; it hyperacetylates the enhancer regions of the Foxp3 gene, enforcing regulatory T cell (Treg) differentiation while concurrently repressing osteoclastogenesis (28, 29). Metabolically, SCFAs directly engage G-protein-coupled receptors (e.g., GPR43/cAMP/PKA signaling axis) to comprehensively re-engineer the oxidative metabolism and clonal expansion of Tregs (30). Furthermore, this synergistic HDAC/GPCR signaling profoundly suppresses pathogenic Th17 generation by dampening AMPK/mTOR and NLRP3 inflammasome pathways (29). Macroscopic nutritional interventions, such as a low-protein diet, have demonstrated remarkable efficacy in mitigating synovial macrophage M1 polarization by activating the NRF2/SIRT3 antioxidant axis, thereby lowering mitochondrial ROS production (40). Concurrently, multi-target botanical regimens (e.g., Fengshining decoction) can proactively reconstruct the intestinal microecology by enriching SCFA-producing probiotics (like Butyrivibrio). This distal intervention reinforces the mucosal barrier and transmits systemic anti-inflammatory signals that successfully suppress the hyperactive HDAC/NF-κB cascade within the remote articular joint (39).

### 6.3 Chronobiology: dietary timing and inflammatory oscillations

A defining, yet historically enigmatic, clinical hallmark of RA is the pronounced circadian rhythmicity of its symptoms, most notably morning joint stiffness. Groundbreaking recent studies have deciphered the metabolic underpinnings of this phenomenon, introducing a novel “dietary timing-microbiota-rhythm” axis (27). The composition and metabolic output of the gut microbiota are not static but exhibit robust diurnal oscillations strictly governed by host dietary timing. Specifically, the temporal intake of nutrients induces circadian fluctuations in the abundance of specific microbes, such as Parabacteroides distasonis. This bacterium utilizes β-glucosidase to enzymatically release glycitein (GLY) from dietary sources in a rhythmic manner. Circulating GLY subsequently enters the joint and modulates the amplitude of synovial inflammation through the SIRT5-NF-κB axis (27). This diurnal metabolic oscillation orchestrates the cyclical rise and fall of pro-inflammatory cytokines, perfectly mirroring the circadian exacerbation of RA symptoms.

### 6.4 Therapeutic implications of the gut-joint conduit

The elucidation of the gut-joint axis elevates RA from a localized joint affliction to a disease of systemic meta-organ cross-talk. Recognizing that intestinal dysbiosis initiates a cascade of NAD+ depletion, HDAC hyperactivity, and rhythmic inflammatory flares provides a powerful rationale for novel therapeutic avenues. Interventions aimed at restoring the systemic metabolic conduit—such as targeted dietary modifications (e.g., high-fiber intake to boost endogenous SCFA production), the administration of SCFA-engineered nano-delivery systems, or chronotherapeutic drug dosing calibrated to microbial metabolic oscillations—hold tremendous promise (29, 30). By replenishing the systemic reservoir of natural epigenetic inhibitors and synchronizing metabolic rhythms, it may be possible to remotely dismantle the pathogenic epigenetic memory locked within the rheumatoid synovium.

## 7 Clinical translation: expanded autoantibody repertoires and metabolic biomarkers

### 7.1 AMPAs as direct pathogenic effectors and the “shared motif” network

The profound metabolic and epigenetic rewiring within the rheumatoid joint does not merely remain confined to the intracellular space; it generates a vast array of modified proteins that spill into the circulation, revolutionizing our understanding of autoantibodies. Crucially, these expanded autoantibodies—the AMPA family, targeting non-canonical, metabolism-derived modifications such as carbamylation, lysine-acetylation, and malondialdehyde (MDA)-acetaldehyde adducts (5, 6)—are not mere epiphenomena or passive bystanders. They are active pathogenic effectors that directly couple humoral immunity to tissue destruction and metabolic rewiring. Recent proteomic studies demonstrate that proteins modified by carbamylation, acetylation, or MDA uniquely bind and activate the classical and lectin complement pathways in an antibody-independent manner, creating an opsonization loop that drastically enhances macrophage phagocytosis and perpetual autoantibody formation (70). Furthermore, B cell clones producing AMPAs exhibit extensive cross-reactivity across different PTMs (e.g., between citrullination and acetylation) through the recognition of structurally analogous “shared motifs,” driving a highly robust and dynamic affinity maturation process (36). Beyond inflammation, specific AMPAs, such as anti-MDA antibodies, directly bind to Fcγ receptors on osteoclast precursors. Crucially, the preferential homing of these circulating autoantibodies to the articular compartment is facilitated by two key factors: the highly permeable neovasculature of the inflammatory pannus, and the localized accumulation of MDA-modified antigens driven by persistent mechanical joint microtrauma and localized oxidative stress. Upon infiltrating the joint space, this specific ligation triggers intracellular HIF-1α and MYC-dependent cascades that force osteoclasts into hyper-glycolysis and accelerated lipid biosynthesis, profoundly accelerating localized bone erosion (71). Thus, the AMPA repertoire functions as a pathogenic bridge transmitting metabolic stress signals into mechanical bone destruction.

### 7.2 Bridging the serologic chasm in “seronegative” RA

Recognizing that these metabolism-driven AMPAs are direct drivers of osteoclastogenesis and complement activation provides a critical breakthrough for clinical diagnostics. Historically, the serological diagnosis of RA has relied predominantly on rheumatoid factor (RF) and anti-citrullinated protein antibodies (ACPAs). However, this binary classification leaves approximately 20–30% of patients diagnosed with “seronegative” RA (SNRA)—a clinically heterogeneous cohort that paradoxically exhibits structural and erosive progression comparable to their seropositive counterparts (6). The discovery of the “PTM multiverse” has revealed that a substantial proportion of SNRA patients actually harbor these unconventional humoral responses against metabolic modifications. Consequently, integrating multiplex AMPA arrays into clinical practice is dismantling the traditional “seronegative” misnomer, allowing for earlier therapeutic intervention before irreversible joint damage occurs.

### 7.3 GlycA: A next-generation window into cardiometabolic risk

While the expanded AMPA repertoire meticulously defines the localized articular autoimmune attack, systemic metabolic biomarkers are urgently needed to capture the whole-body epigenetic and inflammatory tone, particularly to stratify patients for RA’s most fatal comorbidity: accelerated cardiovascular disease (CVD). Traditional acute-phase reactants (like CRP and ESR) often fail to fully capture this underlying cardiometabolic risk. Enter Glycoprotein acetyls (GlycA), a novel composite biomarker quantified via proton nuclear magnetic resonance (1H-NMR) spectroscopy that captures the N-acetyl methyl group signals of circulating acute-phase glycoproteins (72). Unlike transient cytokines, GlycA represents a stable, integrated measure of systemic inflammation and metabolic dysregulation. Recent Mendelian randomization and large-scale cohort studies have firmly established GlycA as a superior, independent predictor of cardiovascular complications and early-onset atherosclerosis in RA, outperforming conventional lipid profiling (33).

Parallel to the diagnostic utility of GlycA, the systemic “lactylation footprint” offers unprecedented prognostic value. Recent multi-omics analyses reveal that pan-lysine lactylation (Pan-Kla) levels in peripheral blood mononuclear cells (PBMCs), alongside specific lactylation-driven gene signatures (such as IKZF1 and LCP1), are significantly elevated in RA patients. Crucially, these peripheral lactylation markers correlate strongly with the severity of immune cell infiltration within the deeply seated synovial tissue, positioning them as highly sensitive, next-generation liquid biopsy targets for early diagnosis and the continuous monitoring of disease activity (73).

### 7.4 Towards precision phenotyping and prognostication

The elevation of GlycA is intricately linked to the hyperactivity of systemic acetylation networks and is notably modifiable by macroscopic environmental factors, such as adherence to the health-promoting EAT-Lancet diet (37). By synergistically deploying multiplex AMPA screening to define the specific PTM-driven autoimmune trajectory, alongside NMR-based GlycA quantification to continuously monitor systemic cardiometabolic vulnerability, clinicians can achieve unprecedented precision in patient phenotyping. This multidimensional profiling transitions RA management from a reactive, one-size-fits-all approach to a proactive, personalized strategy, ensuring that both articular preservation and cardiovascular longevity are addressed (**Table 5**).

**Table 5:** Clinical translation: diagnostics and metabolic-epigenetic restoration.

Clinical Domain	Target/biomarker	Molecular category/agent	Mechanism of action & clinical implication	Ref.
Advanced Diagnostics	AMPAs (e.g., Anti-MDA, AAPA)	Expanded Autoantibody Repertoire	Closes the diagnostic gap in “Seronegative” RA; Anti-MDA directly drives osteoclast glycolysis and bone erosion.	(5, 6, 71)
Advanced Diagnostics	Pan-Kla & Lactylation Genes (IKZF1, LCP1)	Systemic Epigenetic Biomarker (PBMCs)	Correlates strongly with synovial immune infiltration; serves as a highly sensitive, next-generation liquid biopsy target for early diagnosis.	(73)
Prognostic Monitoring	GlycA (Glycoprotein Acetyls)	1H-NMR Systemic Metabolic Biomarker	A highly stable proxy for systemic acetylation; acts as a superior independent predictor for RA-associated Cardiovascular Disease.	(33, 37, 72)
Targeted Therapeutics	Lactate Generation/Efflux	LDHA Inhibitors, MCT1/4 Blockers	Aims to mitigate the synovial “lactate storm,” showing potential to attenuate macrophage M1 polarization and FLS hyper-proliferation.	(8, 15)
Targeted Therapeutics	Epigenetic “Writers” (KATs/NAT10)	Gastrodin, MB-3, Remodelin	Destabilizes KAT8/KAT2A to erase aberrant H3K9la/ac; disrupts NAT10 to halt mRNA ac4C-mediated synovial aggression.	(49, 53, 64)
Targeted Therapeutics	Epigenetic “Erasers” (HDACs/Sirtuins)	Isoform-selective HDACi (Givinostat), Sirtuin Agonists (Resveratrol, NMN)	Rebalances the acetylation network; Sirtuin agonists specifically restore mitochondrial fitness and reverse hyper-acetylated states.	(74–76)
Targeted Therapeutics	Epigenetic “Readers” & Dual-Modulators	I-BET762 (BETi); Catalpol, Cantleyoside	I-BET762 paralyzes matrix-degrading transcription; Catalpol optimizes Treg oxidative metabolism; Cantleyoside enforces FLS apoptosis via AMPK/SIRT1.	(68, 77, 78)
Systemic/Chrono-Therapy	Gut-Joint Axis & Circadian Rhythms	SCFA-delivery, Dietary Timing	Butyrate acts as a natural HDAC inhibitor to restore Th17/Treg balance; timing interventions synchronize inflammatory oscillations.	(27, 29, 30)

## 8 Conclusion and future perspectives: towards metabolic-epigenetic restoration

### 8.1 The paradigm shift from immunosuppression to epigenetic erasure

The management of rheumatoid arthritis stands at the precipice of a monumental paradigm shift. For decades, therapeutic strategies have been dominated by the sequential blockade of terminal inflammatory effectors—cytokines, kinases, and cell surface receptors. However, the persistent “glass ceiling” of refractory disease and rapid post-withdrawal flares underscore a critical limitation: conventional immunosuppressants fail to erase the deeply ingrained “epigenetic scars” residing within synovial cells (9, 32). As elucidated in this Review, the hostile rheumatoid microenvironment acts as a metabolic crucible, generating intermediate metabolites that directly rewrite the chromatin landscape through a highly coordinated “PTM multiverse.” Recognizing that metabolic dysregulation and epigenetic locking are not merely secondary consequences of inflammation, but rather the fundamental engines of disease chronicity (7), opens a revolutionary vanguard for precision medicine: Metabolic-Epigenetic Restoration.

### 8.2 Precision interventions targeting the lactate-lactylation axis

Dismantling the pathological lactate-lactylation axis represents one of the most promising therapeutic frontiers. To relieve the localized “lactate storm,” pharmacological inhibitors targeting the glycolytic rate-limiting enzyme lactate dehydrogenase A (LDHA) or blocking lactate efflux via monocarboxylate transporters (MCT1/4) have demonstrated profound efficacy in reversing macrophage polarization and FLS hyper-proliferation in preclinical models (8, 15). Furthermore, precision intervention at the epigenetic level—targeting the specific “writers” of lactylation—is gaining traction. For instance, recent studies highlight that small molecules, such as the natural phenolic glycoside gastrodin, can directly bind and destabilize the acetyltransferase KAT8. This targeted inhibition has been shown in preclinical models to significantly attenuate aberrant histone H3K9 lactylation (H3K9la), thereby helping to suppress the transcriptional machinery of pro-inflammatory cytokines without causing broad genomic toxicity (49).

### 8.3 Rebalancing the acetylation network: HDACs, KATs, and sirtuins

Restoring the equilibrium between histone acetyltransferases (HATs/KATs) and deacetylases (HDACs/Sirtuins) is equally imperative. While pan-HDAC inhibitors exhibit broad anti-inflammatory effects, their clinical translation in RA is hindered by systemic toxicity. The future lies in isoform-selective HDAC inhibitors (e.g., targeting HDAC1/2 or the class II HDAC inhibitor givinostat) which potently suppress synovial invasion and bone erosion while sparing physiological cellular functions (23, 74, 75). Conversely, suppressing hyperactive acetyl-writers offers another precise angle; for instance, targeting KAT2A via specific inhibitors (e.g., MB-3) or disrupting NAT10-mediated mRNA N4-acetylcytidine (ac4C) modifications significantly curtails NLRP3 inflammasome activation and synovial aggressiveness (53, 64). Parallelly, reactivating the exhausted “epigenetic erasers”—the Sirtuin family (SIRT1/3/6)—holds immense translational potential. Supplementation with NAD+ precursors (e.g., NMN) or the administration of potent naturally derived Sirtuin agonists (such as resveratrol, quercetin, and specific saponins) demonstrated promising potential in *in vitro* and *in vivo* studies to support mitochondrial fitness, mitigate HIF-1α driven angiogenesis, and ameliorate the hyper-acetylated state of key transcription factors like NF-κB and FOXO3a (76, 79–81).

The horizon of metabolic-epigenetic restoration is rapidly advancing toward highly selective molecular tools. Rather than employing broad-spectrum inhibitors that risk genomic toxicity, targeting epigenetic “readers”—such as the bromodomain and extra-terminal (BET) proteins using specific inhibitors like I-BET762—can selectively paralyze the transcription of matrix-degrading enzymes and halt osteoclastogenesis without indiscriminately erasing crucial epigenetic marks (68). Similarly, deploying isoform-selective HDAC1/2 inhibitors effectively dismantles cytokine-driven bone resorption while sparring the physiological functions of non-target cells (82). Within this precision paradigm, natural compounds offer unparalleled multi-target efficacy. Agents like resveratrol and cantleyoside act as SIRT1 modulators, which may contribute to the reduction of glycolysis-fueled pathological angiogenesis and the promotion of FLS apoptosis via the AMPK/SIRT1 axis in experimental models (77, 83). Furthermore, metabolic rewiring of immune cells can be achieved through compounds like catalpol, which promotes Treg differentiation by optimizing oxidative metabolism via the mTORC1/HIF-1α pathway (78). Other complex botanical extracts, such as Wutou decoction and royal jelly acid, directly intercept the “PTM multiverse” by suppressing HMGB1 acetylation and silencing aberrant H3K9 lactylation (84, 85). Together, these metabolism-epigenetic dual modulators herald a highly promising, low-toxicity therapeutic arsenal designed to erase pathogenic cellular memory and achieve sustained remission in RA.

### 8.4 Holistic modalities: the integration of chronotherapy and microbiome engineering

Looking beyond the articular cavity, the future of RA therapy must embrace systemic, meta-organ interventions (86). The newly defined gut-joint axis provides an elegant target for non-immunosuppressive disease modulation. Engineered microbial therapies, including rationally designed probiotic consortia or targeted delivery of short-chain fatty acids (SCFAs) via nanocarriers, can systemically replenish endogenous HDAC inhibitors, thereby remotely repairing the Th17/Treg balance (29, 30). Moreover, the discovery that gut-derived metabolites exhibit robust circadian oscillations dictates the necessity of chronotherapy (27). Synchronizing the administration of metabolic modulators or traditional DMARDs with the host’s natural metabolic and microbial rhythms could drastically maximize drug efficacy while minimizing off-target effects.

In conclusion, rheumatoid arthritis is fundamentally a disease of misdirected cellular memory, sustained by a vicious cycle of metabolic deprivation and epigenetic trapping. The expanding constellation of metabolic-driven PTMs—encompassing acetylation, lactylation, and beyond—provides the molecular cipher to this memory. By integrating high-resolution metabolic profiling (e.g., GlycA) and expanded autoantibody repertoires into early diagnostic frameworks, and by deploying a new generation of metabolic-epigenetic modulators, we possess the tools to intercept the disease at its root. The ultimate therapeutic horizon for RA is no longer merely the continuous pharmacological suppression of symptoms, but the complete epigenetic reprogramming of the synovial niche—a definitive step towards sustained, drug-free remission and functional cure.
